# Assessing Asthma control in UK primary care: Use of routinely collected prospective observational consultation data to determine appropriateness of a variety of control assessment models

**DOI:** 10.1186/1471-2296-12-105

**Published:** 2011-09-29

**Authors:** Gaylor Hoskins, Brian Williams, Cathy Jackson, Paul D Norman, Peter T Donnan

**Affiliations:** 1 Population Health Sciences, School of Medicine, University of Dundee, Mackenzie Building, Kirsty Semple Way, Dundee, DD2 4BF, Scotland, UK; 2 Nursing, Midwifery & Allied Health Professional Research Unit, Iris Murdoch Building, University of Stirling, Stirling, FK9 4LA, Scotland, UK; 3 School of Medicine, University of St Andrews, St Andrews, KY16 9TF, Scotland, UK; 4 School of Geography, University of Leeds, Leeds LS2 9JT, England, UK

## Abstract

**Background:**

Assessing asthma control using standardised questionnaires is recommended as good clinical practice but there is little evidence validating their use within primary care. There is however, strong empirical evidence to indicate that age, weight, gender, smoking, symptom pattern, medication use, health service resource use, geographical location, deprivation, and organisational issues, are factors strongly associated with asthma control. A good control measure is therefore one whose variation is most explained by these factors.

**Method:**

Eight binary (Yes = poor control, No = good control) models of asthma control were constructed from a large UK primary care dataset: the Royal College of Physicians 3-Questions (RCP-3Qs); the Jones Morbidity Index; three composite measures; three single component models. Accounting for practice clustering of patients, we investigated the effects of each model for assessing control. The binary models were assessed for goodness-of-fit statistics using Pseudo R-squared and Akaikes Information Criteria (AIC), and for performance using Area Under the Receiver Operator Characteristic (AUROC). In addition, an expanded RCP-3Q control scale (0-9) was derived and assessed with linear modelling. The analysis identified which model was best explained by the independent variables and thus could be considered a good model of control assessment.

**Results:**

1,205 practices provided information on 64,929 patients aged 13+ years. The RCP-3Q model provided the best fit statistically, with a Pseudo R-squared of 18%, and an AUROC of 0.79. By contrast, the composite model based on the GINA definition of controlled asthma had a higher AIC, an AUROC of 0.72, and only 10% variability explained. In addition, although the Peak Expiratory Flow Rate (PEFR) model had the lowest AIC, it had an AUROC of 71% and only 6% of variability explained. However, compared with the RCP-3Qs binary model, the linear RCP-3Q Total Score Model (Scale 0-9), was found to be a more robust 'tool' for assessing asthma control with a lower AIC (28,6163) and an R-squared of 33%.

**Conclusion:**

In the absence of a gold standard for assessing asthma control in primary care, the results indicate that the RCP-3Qs is an effective control assessment tool but, for maximum effect, the expanded scoring model should be used.

## Background

The ability to identify poor control is a pre-requisite for improving asthma. To achieve the guideline standards for asthma there is a necessity for primary health care professionals to efficiently assess and monitor symptom control. To do this they must utilise questions and tools that are simple and brief to use, easy for the patient to understand, and have a scoring system meaningful to both practitioner and patient. Assessment of asthma control using a standardised tool is now considered good clinical practice [1,2]. There is, however, a lack of empirical evidence on the best tool to use in the primary care setting [3].

The complexity and variability of asthma makes prediction of treatment response difficult [4]. With such a complex disease, no single parameter can accurately classify and assess control in all individuals [5]. To build an accurate clinical picture, a range of criteria have to be assessed based on a patient's symptoms, sleep disturbance, use of rescue medication, daily activity limitation, patient and health professional overall assessment, and lung function. The concern is that patients, and indeed health professionals, often have a different understanding of the language used to describe control [6]. This creates a dilemma for those trying to improve the assessment and management of asthma.

In UK primary care, the tool most commonly used is the Royal College of Physicians 3 Questions(RCP-3Qs) [7]. Conceived as a practical clinical tool that 'makes sense to both clinician and patient' and 'improves standards of care' [7,8], its use has yet to be fully validated. Recently, Thomas et al. (2009) [9] indicated that when using the recommended Asthma Control Questionnaire(ACQ) threshold of 1.0 for 'well controlled' or 'not well-controlled' asthma, an RCP-3Q score of 0 was a good predictor of well controlled asthma but the study was small and the authors conceded that it could not be considered a validation study. There is also an argument that using standardised tools to assess and record relevant information may create a more physician-centred, or template-directed, consultation [10-12].

The UK Quality Outcomes Framework(QOF) rewards practices for use of screening questionnaires in other chronic conditions but not for asthma, despite the availability of recommended measurement instruments. Therefore, the absence of a gold standard screening tool for asthma is a barrier to its inclusion as a QOF outcome measure. This may in turn impact on the consistency of asthma review appointments in the community.

This paper adds to the limited knowledge available on asthma control screening tools by examining the appropriateness of a number of different models of asthma control assessment by:

• Creating models of control from an existing UK data set

• Using statistical modelling to identify the most appropriate model for assessing control.

## Methods

Currently there is no recommended gold standard for assessing asthma control in primary care. Consequently, there is no gold standard against which a measure of asthma control can be assessed. There is, however, strong empirical evidence to indicate those factors which are strongly associated and predictive of asthma control. These include: patient characteristics such as age, weight, presence of symptoms and symptom pattern, as well as factors related to healthcare resource use and organisation and provision of care. As a result, we would expect a good measure of control to be one whose variation was most explained by these factors, and a poor measure to be one which bears little relation to them. Therefore, utilising an extensive UK database, we used multiple regression techniques and statistical tests to examine a variety of current measures (assessing the derived models for fit and performance). We then identified which of them (e.g. GINA, RCP 3 questions) was best explained by the independent variables.

### Recruitment and Data Collection

Between the beginning of January 2001 and the end of December 2005, over 1200 practices from throughout the UK participated in an electronic audit of asthma management. This provided cross-sectional observational review data for more than 78,000 patients from 0 to 99 years of age. The windows-based audit software was informed by the assessment recommendations of the British Thoracic Society (BTS) Guidelines for the management of Asthma and consisted of six data screens. Practice and patient personal details were completed prior to the consultation. The remaining screens, addressing symptoms, inhaler technique, use of self-management plans, compliance, health resource use, education, and therapeutic management, were completed during the consultation. The template acted as an aide memoir for conducting a thorough guideline-based asthma review. Based on previous audit experience [13,14], practices were encouraged to ask their patients about night-day-activity-related symptoms and medication use/compliance in the 4 weeks prior to the review; to observe and record peak expiratory flow rate and inhaler technique at the review; to record ownership and use of a self management plan, need for emergency medication, and health service resource use, since their last review.

For the purpose of the original audit, practice clinical systems were searched for patients > 4 years of age receiving preventative asthma medication BTS treatment step 2 within the previous 12 month period. Systems were also searched for patients who were over-using their β2-agonist medication (more than 6 prescriptions for a short acting bronchodilator in the previous 12 months). Identified patients who met these inclusion criteria were then invited to the practice for a routine review of their asthma. The only exclusion criterion from the study was a co-morbidity of Chronic Obstructive Airways Disease(COPD). The review process was part of normal practice routine. Nurses were not expected to run special sessions in order to undertake the review but were instead required to fit it into their daily work schedule. Those patients who accepted the invitation for a review were asked for written consent for their consultation data to be downloaded anonymously on a monthly basis to a central database at the research centre. Practices signed up to a 12 month audit service and over the course of the year were sent quarterly reports comparing their asthma management with current BTS guidelines. For the purpose of this study data analysis was confined to the information from the first review consultation. In addition, due to the differences in guideline management of children under 13 years of age to adults with asthma [1], only data on patients ≥13 years of age were included in the analysis.

### Data Collection: Additional Data

Prior to analysis the quality of the data was reviewed. Whilst many of the variables were collected as a result of the routine asthma review some were added or enhanced for the purpose of the study. New patient variables were constructed from the existing data. This was then merged with the practice data which had been enlarged by the addition of information about the practice and its geographical area. The final dataset included a Body Mass Index score for each patient and a deprivation indicator for the local area ward in which each GP practice was located [15]. To investigate effects of urban or rural living on asthma control and management, a binary categorisation of rural/urban, [< > 15 persons per hectare (pph) [16]] was attributed to all practices in the dataset. Also added were: Quality Outcome Frameworks points for each practice; distance of the practice to the nearest district general hospital; and the availability of a respiratory specialist within that hospital. The variables used for the multi-level modelling process are itemised in Table 1.

**Table 1:** Variables used for regression modelling.

Patient Variables	
Age	Rescue use inhaled steroid***

Gender	Rescue oral steroid***

Body Mass Index	Emergency Nebulisation***

Active Smoker	Scheduled Consult***

PEFR < 80% predicted/best*	Unscheduled Consult***

Symptoms**	Telephone Consult***

Days Off due to asthma**	Home Visit***

Regularly forget preventer*	Outpatient Visit***

Poor inhaler technique*	A&E visit***

No Self management plan	Admission to Hospital***

Overuse Short Acting Bronchodilator**	Asthma Symptoms** Night/Day/Activity

BTS Treatment Step	

Practice Variables	

Strategic Health Authority (SHA)	Number GPs in practice

SHA Deprivation	Full time (FT) Nurse in Practice

SHA Population	Number of FT Nurses

Primary Care Trust (PCT)	Part time (PT) Nurse in Practice

PCT Deprivation	Number of PT Nurses

PCT Population	Number of Nurses in practice

Ward Deprivation	Way Asthma Care Provided

Ward Population	Presence of Nurse Clinic

Rurality	Nurse with Diploma Level Asthma Training

Practice	Previous Asthma Audit

Practice Population	Previous use of Review Protocol

Full Time (FT) GP in Practice	Asthma UK QOF Points

Number FT GPs	Total UK QOF Points

Part time (PT) GP in Practice	Distance nearest District General Hospital

Number PT GPs	Respiratory Consultant at DGH

* *Assessed at the review consultation*

**In the 4 weeks prior to the review consultation

*** *Since previous review*

Note: Variables used in the construction of a control dependent variable were not used as an independent variable in the regression analysis for that particular control model

### Control Models

**Figure 1:**
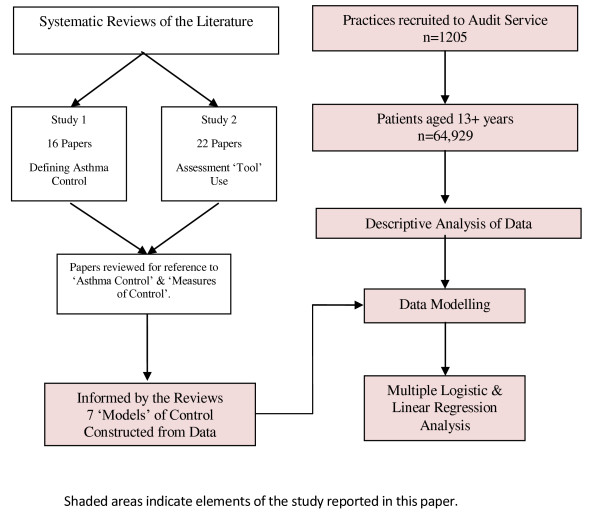
**Flow of the study**.

**Figure 2:**
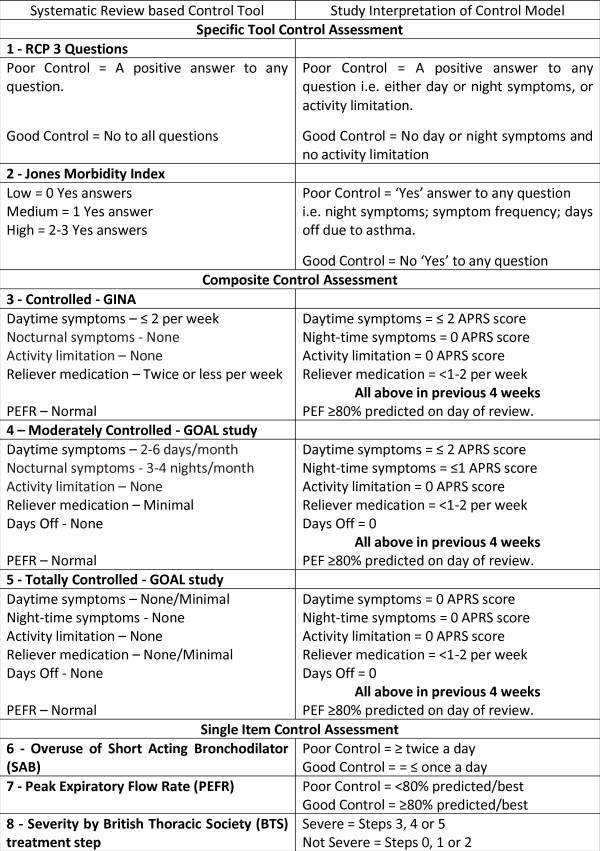
**Asthma control assessment models constructed from the data**.

**Figure 3:**
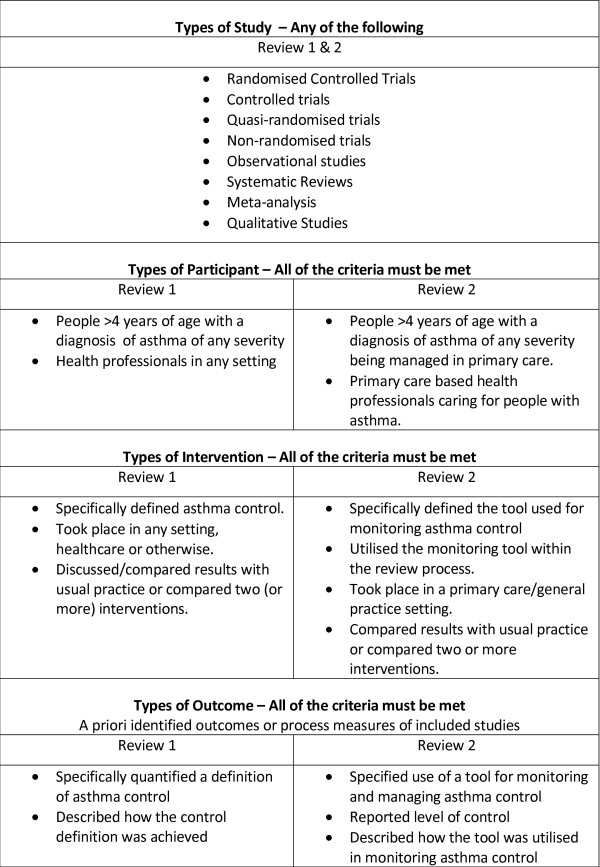
**Inclusion criteria for studies in the systematic review**.

**Figure 4:**
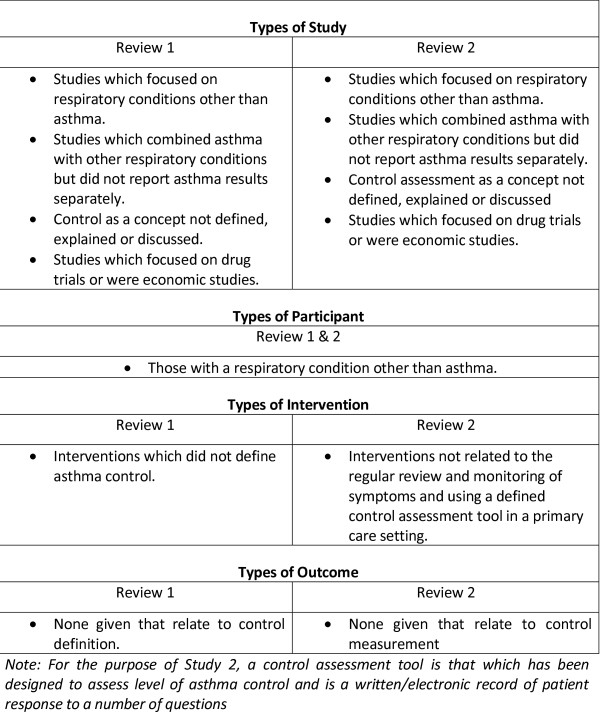
**Exclusion criteria for studies in the systematic review**.

Figure 1 outlines the project process, the shaded area representing the methods and results described in this paper. The dependent binary outcome variable was poor control of asthma. Guided by two systematic reviews of the literature [17] eight binary (Yes = poor control, No = good control) models of asthma control were constructed (Figure 2) from the large UK database. The literature reviews were conducted according to strict methodological criteria (Figures 3 & 4) [18], and were designed to define 'poor' asthma control; and to identify the tools used in primary care to assess asthma control. The reviews identified a total of 26 asthma control assessment tools being used internationally in primary care. However, the range of control models constructed and analysed for this study were restricted by two things:

• The complexity of the scoring mechanism in some of the tools e.g. the Asthma Control Test (ACT) and the Asthma Control Questionnaire (ACQ), made it impossible to model the asthma data to suit the score point for good and poor control.

• Many of the tools were based on the international criteria for good and poor control [2] thus reducing the necessity to model all the individual tools.

**Figure 5:**
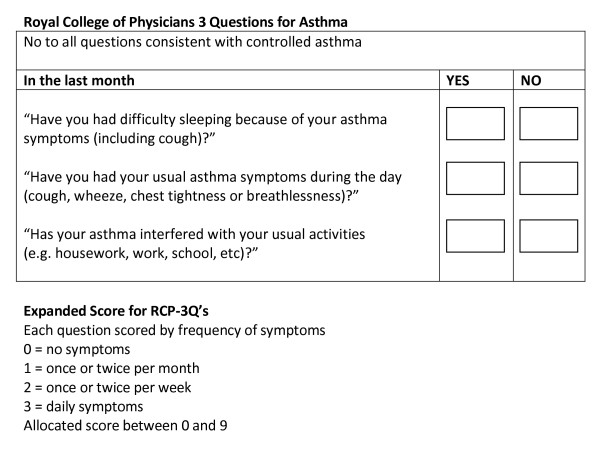
**Royal College of Physicians 3 Questions for Asthma**.

**Figure 6:**
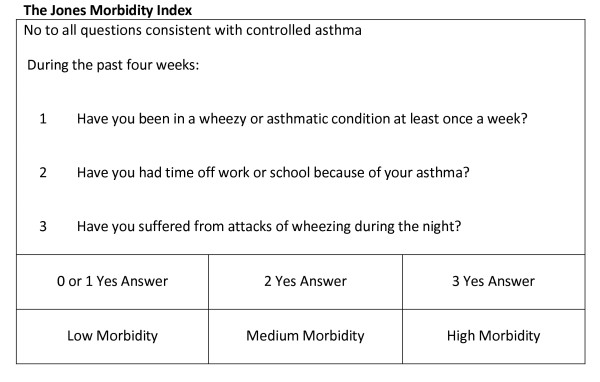
**Jones Morbidity Index**.

Thus, using the information from the literature reviews, the data were modelled to mirror two of the tools recommended by BTS/SIGN guidelines, the RCP-3Qs (Figure 5) and Jones Morbidity Index (JMI) (Figure 6), and three composite measures, one based on the Global Initiative for Asthma(GINA) criteria [2] for controlled asthma, one on the GOAL study [19] criteria for total control, and another on the GOAL study [19] criteria for well/moderately controlled asthma. The GOAL study criteria were based on the GINA definition of asthma control.

**Figure 7:**
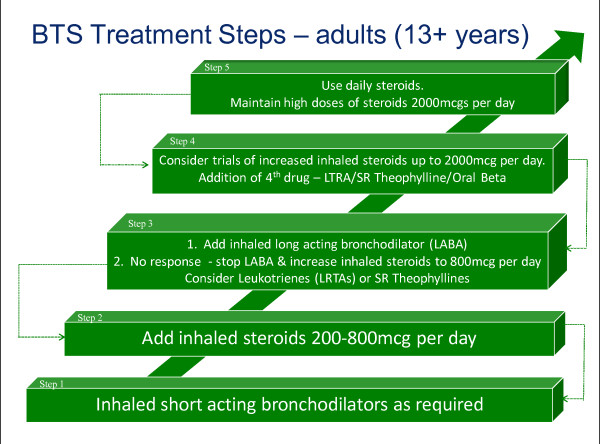
**British Thoracic Society/SIGN Treatment Steps for Asthma (13+ years)**.

Three single component models were also analysed to assess their ability to predict poor control. Peak Expiratory Flow Rate (PEFR) and overuse of bronchodilator medication are important components of a composite assessment tool but have limited ability when used in isolation [3]. This was tested within the analysis. A severity model, based on BTS/SIGN treatment step (Figure 7), was also investigated. Variables used in the construction of each dependent outcome were not used as independent variables in the regression analysis for that particular control model. Based on the findings from the logistic regression, linear regression was also carried out on an expanded score model of the RCP-3 Questions (Figure 5). Scored by question and frequency of symptoms [0(no symptoms) to 3(daily symptoms)], each patient was allocated a score between 0 and 9.

### Statistical Analysis

Utilising the UK database descriptive statistics using Chi-squared tests for categorical data and t-tests for continuous data were carried out on the baseline data using SPSSv15(SPSS Inc., 2006). The Chi-squared statistic was reported for comparisons of categorical data and the Chi-Squared test for trend reported where significant.

Multiple regression modelling, accounting for practice clustering of patients, was undertaken to determine the strength of association of the independent variables (Table 1) within each model. Multiple logistic regression multi-level modelling was used to derive the eight simple binary (yes/no) models using STATAv9(Stata Corp LP, 2007). Linear regression analysis was used to analyse the RCP-3Q expanded score model. Model fit for all logistic regression models was assessed using three tests-Area Under the Receiver Operating Characteristic (AUROC) (c-statistic) which measured the ability of the model to predict control; Pseudo R-squared which was used to assess the amount of explained variance within the model; and Akaikes Information Criteria (AIC) used to assess fit and performance. The linear regression model was assessed using R-squared and AIC. Using these measures we ranked the performance of all eight models.

### Ethical Approval

Ethical approval for the data collection was given by the Regional Medical Research Ethics Committee.

## Results

From the 10,432 primary care practices in the UK (correct at time of data collection), 1,205 practices of all sizes and with a wide geographical spread, submitted usable practice data and data on 64,929 patients who attended for a routine asthma review. The study practices represented almost 12% of all UK practices. The proportion of practices from England, Scotland and Wales was similar (Table 2). Northern Ireland was considerably lower.

**Table 2:** Participating practices by UK country

	Total UK Practices	Study Practices	Proportion
England	8,551	1,014	11.9%

Scotland	1,014	116	11.4%

Wales	502	55	11.0%

Northern Ireland	365	20	5.5%

UK Total	10,432	1,205	11.6%

**Figure 8:**
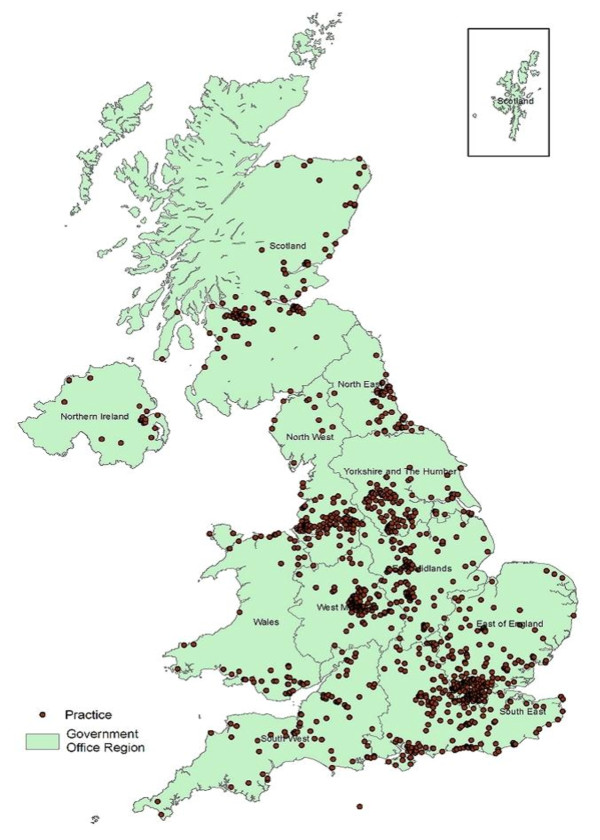
**UK distribution of study practices**.

Study practices were spread over all 28 English Strategic Health Authorities (SHA); 11(73%) of the 15 Scottish Health Boards; 16(73%) of the 22 Health Authorities in Wales; and all four in authorities in Northern Ireland (Figure 8). Practices were situated in 305(89%) of the 344 UK Primary Care Trusts (or equivalent e.g. Community Health Partnership (CHP) in Scotland).

The PCTs represented in the study had a higher mean population size than the average for all UK PCTs [Study Mean 220,940 (Upper Quartile(UQ) 247,996; Lower Quartile(LQ) 130,180) v National Mean 170,872(UQ 207,796; LQ 112,355)], indicating a slightly higher number of urban or semi urban practices in the study cohort. However, over 30% of the practices were situated in rural or semi-rural areas (Table 3).

**Table 3:** Practice Baseline Characteristics (n = 1205)

		Missing
Rurality: Urban practices	826 (68.5%)	0 (0.0%)

Practice population size	Mean 6,862	5 (0.4%)
	Lower Quartile 3,877.5	
	Upper Quartile 9,000	
	Range 367 to 32,000	
	Median 6,300	

Practices with a: Full time GP	1,090 (90.5%)	91 (7.5%)
Part time GP	381 (31.6%)	91 (7.5%)
Full time nurse	787 (65.3%)	105 (8.7%)
Part time nurse	478 (39.6%)	105 (8.7%)

Deprivation Score	Mean 1.65 (SD 3.62)	1 (0.08%)
	Range -5.21 to 13.21	
	Median = 1.18	

Distance of practice from the nearest DGH	Mean 4.86 miles (SD 5.71)	1 (0.08%)
	Range 0 to 96	
	Median = 3	

Respiratory Specialist in-situ in nearest DGH	1,150 (95%)	35 (2.9%)

Practices who had:		
Nurse run asthma clinic	830 (68.9%)	68 (5.6%)
Nurse with an accredited Asthma Diploma	897 (74.4%)	48 (4.0%)
Carried out asthma audit in the previous 3 years	723 (60.0%)	55 (4.6%)
Previous use of Asthma Stamp (Protocol)	561 (46.6%)	91 (7.5%)
Achieved ≥70% of Asthma QOF* Points 2004/5	617 (51.2%)	22 (1.8%)
Achieved ≥70% of Total QOF* Points 2004/5	1,152 (95.6%)	19 (1.6%)

QOF = Quality Outcome Framework

DGH = District General Hospital

Comparison of the mean local area deprivation score [15] for the study cohort [Mean 1.6508 (UQ 3.8750; LQ -1.1900)] with that for all UK practice wards [Mean 0.0009 (UQ 2.0848; LQ -2.6114)] confirmed that the study cohort was skewed slightly towards practices in more deprived areas, probably because of the higher number of urban practices in the study.

Practice size ranged from very small (367) to very large (32,000) (Table 3). Eight hundred and forty one (70%) of the practices had an asthma register of between 5 and 10% of their total patient population.

Patient characteristics and asthma control status are summarised in Table 4. The mean Body Mass Index (BMI) of the study population was 26.16 (SD 5.79). Thirty nine percent of patients (25,537) were within normal BMI range (18 to 25). Nearly 54% were overweight or obese: 33% (21,546) were overweight (BMI 25 to 30); 21% (13,373) obese (BMI > 30). The proportion of current smokers [13,531(21%)] was slightly lower than the UK rate of approximately 1 in 4 adults [20].

**Table 4:** Baseline characteristics for patients aged 13 years and over (n = 64,929)

Issues assessed/discussed at consultation	Number	%
Age Mean (SD)	46.9 (SD 19.5)	

Patient numbers by age band: 13-19 years	7,047	11

20-29 years	7,258	11

30-39 years	10,616	16

40-49 years	10,029	15

50-59 years	10,298	16

60-69 years	10,259	16

70+ years	9,422	15

Female	37,826	58

Body Mass Index (BMI) Mean (SD)	26.16 (SD 5.79)	

Patient numbers by BMI band: ≤18	4,473	7

> 18-25	25,537	39

> 25-30	21,546	33

> 30	13,373	21

PEFR at consultation < 80% predicted	1,971	3

Days off in last 4 weeks due to asthma	8,382	13

Smoker	13,531	21

Symptoms in last 4 weeks	54,503	84

Inhaler Device(s) Technique observed as poor	6,789	10

Inhaler occasionally forgotten/not taken	18,869	29

Self management plan not in use prior to consultation	39,938	62

Temporary Increase in Inhaled Steroid since last review	3,157	5

Rescue Oral Steroid since last review	3,223	5

Emergency Nebulisation since last review	1,528	2

Over use of Inhaled Short Acting Bronchodilator*	30,360	47

Scheduled consult since last review	2,626	4

Unscheduled consult since last review	1,755	3

Telephone consult since last review	321	0.5

Home visit since last review	158	0.2

Outpatient visit since last review	95	0.1

A&E visit since last review	292	0.4

Admission to Hospital since last review	58	0.1

BTS Treatment Step prior to review: BTS Step 0	5,477	8

BTS Step 1	7,274	11

BTS Step 2	29,629	46

BTS Step 3	10,678	16

BTS Step 4	11,491	18

BTS Step 5	380	1

*Overuse of Short Acting Bronchodilator = SAB inhaler required more than once every day in the four weeks prior to the consultation

BTS = British Thoracic Society

**Figure 9:**
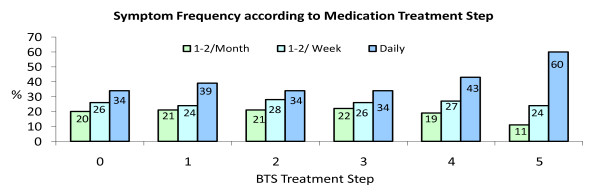
**Symptom frequency according to BTS medication treatment step**.

At the time of the review 54,503 (83.9%) patients reported experiencing some degree of asthma related symptoms in the immediate 4 week period preceding the consultation: 13,438 (20.7%) on one or two days in the month; 17,451 (26.9%) patients one or two days a week; and 23,614 (36.4%) on a daily basis. Four out of five patients [52,178 (80%)] were on preventative asthma medication (BTS treatment steps 2-5) prior to the review. Although, as one would expect, level of treatment step was significantly associated to symptom reporting (Table 5), patients on all levels of treatment were experiencing frequent symptoms (Figure 9).

**Table 5:** Level of reported symptoms within each treatment step

BTS Treatment step	0 n = 5,477	1 n = 7,274	2 n = 29,629	3 n = 10,678	4 n = 11,491	5 n = 380
Symptoms	4,401 80%	6,065 83%	24,801 84%	8,754 82%	10,125 88%	357 94%

A Chi squared test for trend showed a statistically significant difference in distribution: λ^2 ^trend 146.811 df 1 p < 0.0001

Prior to the review 30,360(47%) patients were reportedly overusing their reliever inhaler (more than 6 prescriptions for a short acting bronchodilator in the previous 12 months), with males statistically more likely than females to be using it more than once every day [M 12,938(48%) v F 17,422(46%); p < 0.0001 (OR 0.93(95%CI 0.91-0. 96)]. Overuse increased with age, rising from 2,361(33.5%) of patients in the 13 to 19 year age band to 4,777(51%) of patients in the 70+ age range (p < 0.0001). It was also associated with weight being greatest in those with a BMI over 30 [6671(50%) of 13,373 patients] and lowest in those with a BMI of 18 to 25 [11,521(45%) of 25,537 patients] (p < 0.0001). Reported overuse of inhaled reliever medication was also linked to level of preventive medication treatment step with patients on low or no preventative medication more likely to overuse [Steps 0-2: 19,446(46%) v Steps 3-5: 10,914(36%); p < 0.0001 (OR 0.90(0.88-0.93)].

There was also a significant difference in the level of poor compliance between:

• Gender-males being more likely than females to forget their inhaler [M 8,672(32%) v F 10,197(27%); p < 0.0001 (OR 1.27(1.23-1.32)].

• Medication treatment step-patients on treatment step 0, 1 or 2 being more likely to forget to take their inhaler than patients on steps 3, 4 or 5 [Steps 0-2: 14,495(34%) v Steps 3-5: 4,374(19%); p < 0.0001 (OR 2.16(2.08-2.25)].

• Age-teenagers and young adults being more likely to forget to take their preventer inhaler [13-19 yrs: 2,890(41%); 20-29 yrs: 2,905(40%); 30-39 yrs: 4,029(38), 40-49 yrs: 3,271(33%), 50-59 yrs: 2,426(24%), 60-69 yrs: 1,888(18%); and patients aged 70+yrs: 1,460 (15.5%) (p < 0.0001)].

• BMI-the more overweight a patient the less likely they were to forget their preventer inhaler (BMI < 18: 1,501(34%); 18- < 25: 7,606(30%); 25-30: 6,108(28%); 30+: 3,654(27%), (p < 0.0001).

### Regression analysis of Asthma Control Models

Of the eight binary control models investigated, the RCP-3 Questions provided the best fit statistically. It had the second lowest AIC, highest pseudo R-squared (amount of explained variability) of 18% and an AUROC of 0.79 (Table 6). An AUROC of 0.79 is on a par with, for example, the Framingham predictors of Coronary Heart Disease [21]. By contrast, the composite model based on the GINA definition of controlled asthma had a higher AIC, an AUROC of 0.72, and only 10% variability explained. The PEFR model had the lowest AIC, an AUROC of 71% and only 6% of variability explained.

**Table 6:** Comparison of performance of multiple regression models

Control Model	Pseudo R-squared	Akaike's Information Criterion	Area Under ROC Curve
RCP-3 Q Model	0.1803	46,933	.7924 95% CI 0.7963, 0.7885

Jones Morbidity Index	0.1352	77,811	.7407 95% CI 0.7444, 0.7370

GINA Control	0.0977	61,868	.7135 95% CI 0.7180, 0.7090

GOAL Total Control	0.0843	51,660	.7028 95% CI 0.7079, 0.6969

GOAL Well Controlled	0.0845	65,481	.6970 95% CI 0.7015, 0.6925

PEFR	0.0615	16,602	.7085 95% CI 0.7183, 0.6985

Overuse of SAB	0.0833	82,299	.6942 95% CI 0.6983, 0.6901

BTS Treatment Step	0.0677	78,223	.6711 95% CI 0.6756, 0.6665

Linear Regression	R-squared	AIC	

RCP-3 Q Expanded Score Model	0.3258	28,616	N/A

The Royal College of Physicians 3 Question model was interrogated further to investigate whether expansion to include a score for the various symptom frequency levels within each of the three questions contributed to a more robust assessment tool. Compared with the RCP-3Q binary model the RCP-3Q expanded score model provided the best fit with a lower AIC (28,6163) and an R-squared of 33% (Table 6).

## Discussion

Statistical modelling found the Royal College of Physicians 3 Questions for asthma was the 'best fit' of the control models tested for determining asthma control in a routine review consultation. However, to be effective, a simple scoring system should be incorporated into the assessment process. The results may prove useful when determining future support strategies for UK Primary Care.

### Strength of the Study

The difficulties observed in conducting randomised control trials in primary care means that the results from good quality observational data need to be used for planning and developing health care policy. Producing a predictive model requires data representative of the general asthma population. The practices that provided data mirrored the distribution pattern of practices throughout the UK and thus were representative of-urban, semi-urban, semi-rural and rural area practices. They ranged in size and represented a wide range of social deprivation from highly deprived to highly affluent. With all levels of deprivation strongly represented the presence of slightly more deprived practice ward areas in the dataset was in actual fact a strength of the study as it ensured that any representation was in favour of those areas where there is potentially poorer control of asthma. This is preferable to over-representation of more affluent areas where control may be better [22,23].

The patients were representative of genders, all ages, and the full range of asthma severity. The number of patients on individual practice asthma registers was consistent with nationally expected numbers of asthmatic sufferers within a practice [24]. The greater number of women (F 58% v M 42%) in the study cohort is indicative of both the higher rate of asthma in adult females, particularly in the older age groups [25], and the tendency for women to be more likely to attend for a review [26]. The proportion of current smokers within the dataset was only slightly lower than the UK smoking rate and was most likely due to the fact that the patients in the study were all suffering from a respiratory disease.

The decision to create a categorical variable was a direct reflection of the structure of many of the control assessment tools in use. Although control is on a continuum from mild to very severe it is apparent that a desired feature from a clinical viewpoint should be a distinct point at which poor control is considered a possibility. It should be emphasised that the assessment questionnaires are not meant to be used as stand-alone tools, but act as a guide to a more thorough review.

The data was from a very large representative cross section of the UK's primary care asthma community. In addition, the regression techniques and goodness-of-fit measures used to test the data ensured that the results could be considered at least internally valid. The use of AUROC, AIC, Pseudo R-squared, and R-squared, allowed comparison of model performance by ranking the outcome. The use of statistical testing to support the findings from cross-sectional observational data is an important step forward in finding the evidence required for supporting change [27].

### Study Limitations

Use of a tool in the form of a questionnaire to aid assessment of asthma can provide clinicians with confidence that they will be able to identify patients with sub-optimal control [3]. The questionnaire must be simple, brief, easy to use, patient centred, and suitable for use in every consultation about asthma [7,28]. The British asthma guideline gives examples of a number of tools which may be appropriate, one of which is the Royal College of Physicians 'Three Key Questions' (RCP-3Q) [7]. The GINA guidelines [2] suggest several alternate control assessment tools such as the Asthma Control Test [29]; Asthma Control Questionnaire [30]; Asthma Therapy Assessment Questionnaire [31]; and the Asthma Control Scoring System [32]. Both guidelines stop short of recommending any one particular tool for use in the primary care setting. The disparity in their 'recommendations' highlights the level of uncertainty that still exists on the best method for use in primary care [3].

Care must therefore be taken when making conclusions based on cross-sectional data from an observational study. This study was not designed to test the RCP-3Qs but to look at how poor control was being defined and operationalised in primary care and to test, where possible, the strength of each assessment model for predicting poor control of asthma. The data analysis was informed by the literature reviews and utilised all the control assessment sources possible for the available data. The number of control models tested was restricted by the structure of some of the other available tools e.g. the ACQ or the ACT, and by the confines of the existing data set. The ACQ and the ACT have more complex scoring systems not easily deconstructed and replicated for this analysis [30,33-35]. This limited the number of control models that could be tested. However, it was clear from the literature that many researchers were basing their control assessment measures on recommendations from published guidelines. It seemed appropriate therefore to construct a model based on the most commonly used criteria in the papers reviewed, the GINA criteria for control [2]. The two additional composite models of well/moderate control and total control, based on the study by Bateman et al. [19], were also constructed on this premise.

The simplicity of the RCP-3Qs meant that the model created for the study was a direct reflection of the tools properties (one yes answer being suggestive of poor control). The Jones Morbidity Index on the other hand was less clear cut, the tool indicating three levels of morbidity (low, medium and high), and a decision was made to use the medium morbidity level (1 Yes answer) to define poor control [36]. A second model to capture only the patients who fell into the more stringent severity classification (2 or 3 Yes answers) may have resulted in a better performing model but the decision to use the less stringent measure was in line with the parameters for control in the RCP-3Q model. For both models, ability to measure control effectively may be dependent on the question to which the one 'Yes' answer refers.

The available evidence indicated that single component measures are not effective for identifying poor control [3,37]. The inclusion of two single item models in the analysis provided the opportunity to test this against both composite models of control measurement and morbidity question models.

As we were looking at models that predicted control it was necessary to include patients with both poor and good control. The search criterion given to practices to assist them with the procedure for identifying patients for review was a pragmatic solution to facilitate organisation of the invitation process. However, the consequence of this guidance could have resulted in the over-inclusion of patients at high risk of having poorly controlled asthma, thus compromising the generalisability of the study findings. The inclusion of patients on all medication steps, and with a range of frequency and type of control issue, indicates that the study cohort transcended the range of asthma patients managed in primary care. The number of patients who reported control issues is, in fact, comparable with those reported by other studies [38-40].

### Implications for practice

It is important for health professionals to accurately assess the level of asthma control experienced by their patients. Using a specific tool to aid this process not only increases their ability to identify sub-optimal control [3], but provides a platform for improving communication [41,42]. The ACT is used widely in the international primary care setting and has been tested against the GINA standards [43-45] however the lack of a recommended gold standard assessment tool for use in primary care has meant that the RCP-3Qs remains the most commonly used tool in the UK. Use of the RCP-3Qs on every occasion and by every health professional who sees the patient for their asthma not only has implications for the way control is assessed but has potential for increasing confidence in the management process.

This work, along with Thomas et al. [9], is a timely addition to the small amount of available evidence supporting the ability of the RCP-3Qs to assess asthma control. Health professionals are constantly asked to prove the care they provide is effective. The original concept of the RCP-3Qs was to develop a health outcome indicator for the assessment of asthma morbidity that could be used in both primary and secondary care. However, the lack of validated evidence of the 'tool's' ability to accurately measure asthma control has limited its use. This study provides additional evidence that, in the absence of a gold standard definition of control and assessment method, the RCP-3Q tool has the ability to identify poor control in patients with asthma.

## Conclusion

Practical guidance on the best method to monitor and assess asthma control is required. The study underpinning this work was conducted on a large number of patients with a wide range of asthma severity and control, and from all levels of social strata across the UK. It supports the conclusions of Thomas et al. [9] that, along with other assessment tools such as the ACT, the RCP-3Qs can be used with confidence in UK primary care when reviewing people with asthma. It is quick and easy to complete and can be used with or without a scoring system, although the latter is more sensitive to poor control. The results may prove useful when determining future support strategies for UK Primary Care. Until there is evidence to negate its use in favour of another tool its use in UK primary care should be supported.

## Conflict of interest

The authors declare that they have no competing interests.

## Authors' contributions

GH managed the data collection, prepared the datasets, analysed the data, and wrote the first draft of the paper. PD, BW and CJ supervised and advised on the design of the project. In addition PD directed the statistical analysis; BW contributed to the focus of the analyses and interpretation of the data; and CJ advised on the clinical interpretation of the data. PN assisted with data preparation by geo-coding practice locations and linking them to primary care organisation geographies. He also obtained census data and adjusted these to Primary Care Organisation geographies and then calculated the deprivation scores and population densities which were used in analyses. All authors read and approved the final draft.

## Pre-publication history

The pre-publication history for this paper can be accessed here:

http://www.biomedcentral.com/1471-2296/12/105/prepub
